# Cytotoxicity of TSP in 3D Agarose Gel Cultured Cell

**DOI:** 10.1371/journal.pone.0128739

**Published:** 2015-06-09

**Authors:** Song-I Chun, Chi-Woong Mun

**Affiliations:** Department of Biomedical Engineering / u-HARC, Inje University, Gyeongman, Republic of Korea; Chang Gung University, TAIWAN

## Abstract

**Purpose:**

A reference reagent, 3-(trimethylsilyl) propionic-2, 2, 3, 3-d4 acid sodium (TSP), has been used frequently in nuclear magnetic resonance (NMR) and magnetic resonance spectroscopy (MRS) as an internal reference to identify cell and tissue metabolites, and determine chemical and protein structures. This reference material has been exploited for the quantitative and dynamic analyses of metabolite spectra acquired from cells. The aim of this study was to evaluate the cytotoxicity of TSP on three-dimensionally, agarose gel, cultured cells.

**Materials and Methods:**

A human osteosarcoma cell line (MG-63) was selected, and cells were three dimensionally cultured for two weeks in an agarose gel. The culture system contained a mixture of conventional culture medium and various concentrations (0, 1, 3, 5, 7, 10, 20 30 mM) of TSP. A DNA quantification assay was conducted to assess cell proliferation using Quant-iT PicoGreen dsDNA reagent and kit, and cell viability was determined using a LIVE/DEAD Viability/Cytotoxicity kit. Both examinations were performed simultaneously at 1, 3, 7 and 14 days from cell seeding.

**Results:**

In this study, the cytotoxicity of TSP in the 3D culture of MG-63 cells was evaluated by quantifying DNA (cell proliferation) and cell viability. High concentrations of TSP (from 10 to 30 mM) reduced both cell proliferation and viability (to 30% of the control after one week of exposure), but no such effects were found using low concentrations of TSP (0–10mM).

**Conclusions:**

This study shows that low concentrations of TSP in 3D cell culture medium can be used for quantitative NMR or MRS examinations for up to two weeks post exposure.

## Introduction

Nuclear magnetic resonance (NMR) spectroscopy can provide detailed insights into the molecular properties of both liquids and solids, including chemical structures and the dynamic changes that result in chemical shifts [1]. NMR and MRS have been widely used to elucidate the structure of chemical metabolite compounds and proteins [2, 3]. Magnetic resonance (MR) imaging provides excellent soft tissue differentiation and allows for *in vivo* assessment of the physiologic and metabolic properties of tissue [4, 5]. Metabolites are substances produced by metabolic processes, and their characterization can provide insights into the mechanisms by which genomic and environmental factors affect metabolic processes. A quantitative analysis of tissue metabolites using NMR spectroscopy provides an important source of information about the stereochemistry of the analyzed substrates [3]. A chemical shift is defined as the difference in resonant frequency compared to a reference signal. Tetramethylsilane (TMS), 4,4-dimethyl-4-silapentane-1-sulfonic acid (DSS) and 3-trimethylsilylpropionic acid (TSP) have been extensively used as internal chemical shift standards[3, 6–8]. Although metabolomic analysis of tissues is widely applied in fields such as medicine, toxicology, and environmental science, a thoroughly validated method for sample preparation is lacking [9].

A number of investigators have been attempting to obtain spectral information on cells and tissues in *in vitro* environments. To assess metabolites at the cellular level, invasive methods such as lysing the cells and extracting the internal material, or acquiring signals in harsh survival conditions (eg., samples in NMR tube) are used for NMR examinations [7, 9–11]. To obtain a high-quality spectrum, the stability and homogeneity of the magnetic field during acquisition are essential. NMR spectrometers attempt to correct the drift of the magnetic field as it occurs to maintain the stability of the field, this is termed “the lock system” [12]. A deuterium (^2^H as D_2_O) NMR signal is used to lock the magnetic field during an experiment by ensuring that the frequency of the ^2^H NMR signal remains constant.

Kwak et al. reported previously that the locking solvent (D_2_O) is cytotoxic. MG-63 cells (Human osteosarcoma cell line) cultured three-dimensionally in alginate beads with either 40% or 100% D_2_O, for 3 h and 24 h, had reduced viability [13]. TSP concentrations of 0, 0.3 and 1.0 mM did not significantly affect the viability of MG-63 cells. These results show that for living cell studies, weak TSP solutions (concentrations less than or equal to 1.0 mM) can be used as a reference material, but that locking solutions containing more than 40% D_2_O should not be used. One study quantitated cell and tissue metabolites from identical specimens during culture [14]. Since repetitively measuring the metabolites from identical cell specimens is important for determining the effects of TSP, we measured the effects of TSP (>1.0 mM) on cells during long exposure periods (>24 h). In the present study, cell proliferation and viability were examined for two weeks using various TSP concentrations (up to 30 mM) as a reference reagent for ^1^H NMR. Conventional NMR studies discard the cell specimens after the course of the examination due to the cytotoxic procedures required, such as specimen preparation, cell lysis and extraction of cellular material [1, 9, 10]. These methods require a large number of disposable specimens for each repeated experiment, and are therefore, inevitably, time-consuming and costly. The appropriate concentration and the cytotoxicity of ^1^H NMR spectroscopy internal standards, mixed with culture medium for long-term cell culture, have not been determined.

In this study, the cytotoxic effect of various TSP concentrations (1 to 30 mM) on MG-63 cells, three-dimensionally cultured in agarose gels for two weeks, was evaluated by measuring cell proliferation and viability.

## Materials and Methods

### Cell Preparation

A human osteosarcoma cell line (MG-63, KCLB No.21427, Korean Cell Line Bank) was used to evaluate the cytotoxicity of 3-(trimethylsilyl) propionic-2, 2, 3, 3-d4 acid sodium salt (TSP, Sigma-Aldrich Co. LLC., USA). High Glucose Dulbecco’s Modified Eagle’s Medium (DMEM-HG, Gibco, Invitrogen Co., USA) containing 10% fetal bovine serum (FBS; Hyclone Laboratories, Inc., USA), and 1% antibiotics (100 IU/ml penicillin, 100 μg/ml streptomycin), was used as the culture medium. Cells were incubated at 37°C in an atmosphere of 95% air and 5% CO_2_. The medium was replaced every 3–4 days.

### Two-dimensional Cell Culture

MG-63 cells were isolated using 0.05% Trypsin/EDTA and centrifuged at 1500 rpm for 3 min. Isolated cells, 1 × 10^4^ cells/ml, were seeded at 4-well plate. The cells were then cultured using DMEM-HG medium containing 10% FBS and 1% antibiotics. The prepared cells were divided into eight groups of the following TSP concentrations: 0, 1, 3, 5, 7, 10, 20, and 30 mM. The cells were incubated at 37°C in an atmosphere of 95% air and 5% CO_2_ for 7 days, and the medium was replaced every 3 days. Proliferation and apoptosis of the cultured cells were evaluated by TUNEL assay/Hoechst staining. The TUNEL assay was performed In Situ Cell Death Detection Kit (Roche, Laval, QC, Canada). The cells were fixed with a freshly prepared fixation solution (4% paraformaldehyde in PBS, pH 7.4) for 20 min at room temperature. After cell incubation with freshly prepared permeabilization solution (0.1% Triton X-100) for 15 min, the cells were labelled using the TUNEL reaction mixture in a humidified atmosphere for 60 min at 37°C in the dark. The cells were subsequently rinsed three times with PBS to stop the reaction and nuclei were stained with Hoechst 33258 (Life technologies, USA) for 15min in the dark. Samples were directly analyzed using a fluorescence microscope.

### Three-dimensional Cell Culture

MG-63 cells were isolated as previously described. The cell of seeding density 2 × 10^5^ cells/ml, were then immobilized in a 1% agarose gel (Low gelling temperature Agarose, Sigma, USA). A portion of the cell suspension containing 1 × 10^5^ cells/500 μl was added to an equal volume of sterilized, and molten (37°C), 2% (w/v) agarose in phosphate-buffered saline (PBS; Sigma, USA) solution [15, 16]. A 500-μl aliquot of the mixture was then loaded into a 24-well plate and kept at room temperature for 30 min to allow the gel to solidify. The cells were then cultured using DMEM-HG medium containing 10% FBS and 1% antibiotics. The prepared cells were divided into eight groups of the following TSP concentrations: 0, 1, 3, 5, 7, 10, 20, and 30 mM. The cells were incubated at 37°C in an atmosphere of 95% air and 5% CO_2_ for 14 days, and the medium was replaced every day.

### Cell Proliferation

DNA quantification was performed to assess cell proliferation using the Quant-iT PicoGreen dsDNA reagent and kit (Molecular Proves, Eugene, OR, USA). Fluorescent expression of PicoGreen dye binding to cellular nucleic acids was used to quantify DNA. After culturing for 1, 3, 7 and 14 days, cells encapsulated in agarose gel were directly digested in papain buffer without any prior extraction. The cell samples were placed at 60°C for 24 h in 1.5-ml sterile Eppendorf tubes with 1 mL of 0.2-μm filter-sterilized, papain digestion buffer (5 mM L-cysteine, 100 mM Na_2_HPO_4_, 5 mM EDTA, pH 7.5), containing 125 μg/ml papain type III. Following digestion, samples were heated to 70°C for 10 min to melt the agarose, vortexed, and then centrifuged at 21,000 g for 1 min at room temperature. The supernatant (100 μl) was then transferred to a 96-well plate and 100 μl of PicoGreen fluorescence reagent (1:200 in Tris-EDTA buffer, Invitrogen Co., Grand Island, NY, USA) were added [13]. DNA standards were prepared using the same procedure. Fluorescence was measured in a multi-detection reader (Synergy HT BioTek, Winooski, VT, USA) and sample values were calculated using the known DNA standards.

### Cell Viability

Cell viability in agarose gel was measured using a Live/Dead viability/cytotoxicity Kit (Molecular Probes, Invitrogen), using the same cell preparation and incubation times as in the previously described proliferation assay. The medium was removed from the cells, and 500 μl of dye reagent, containing 1 μM calcein and 2 μm ethidium homodimer-1, were added to the top of the agarose gel. The agarose gel samples were then incubated at room temperature for 30 min, and cross-sectional images were obtained using a fluorescence microscope [15]. Cell viability (%) was calculated using Eq (1). The number of living and dead cells was estimated by counting the living (green), and dead (red), pixels in the obtained fluorescence images using MATLAB (R2008a, Mathworks, USA).

$$Cell\;Viability(\%)=\frac{Living\;Cells}{Living\;Cells+Dead\;Cells}\times 100$$ (1)

### Statistical Analysis

Three independent experiments were performed and data were obtained from five samples for statistical analysis. All results are presented as means ± standard deviation. Statistical analysis was performed using the Statistical Program for the Social Sciences, version 19.0 (SPSS, IBM Co., monk, NY, USA). One-way ANOVA tests were conducted to examine the cytotoxic effects of TSP using a significance level of p < 0.05 with a *post hoc* Dunnett’s t-test.

## Results

### TSP’s Cytotoxicity in 2D Cultured Cell

**Fig 1:**
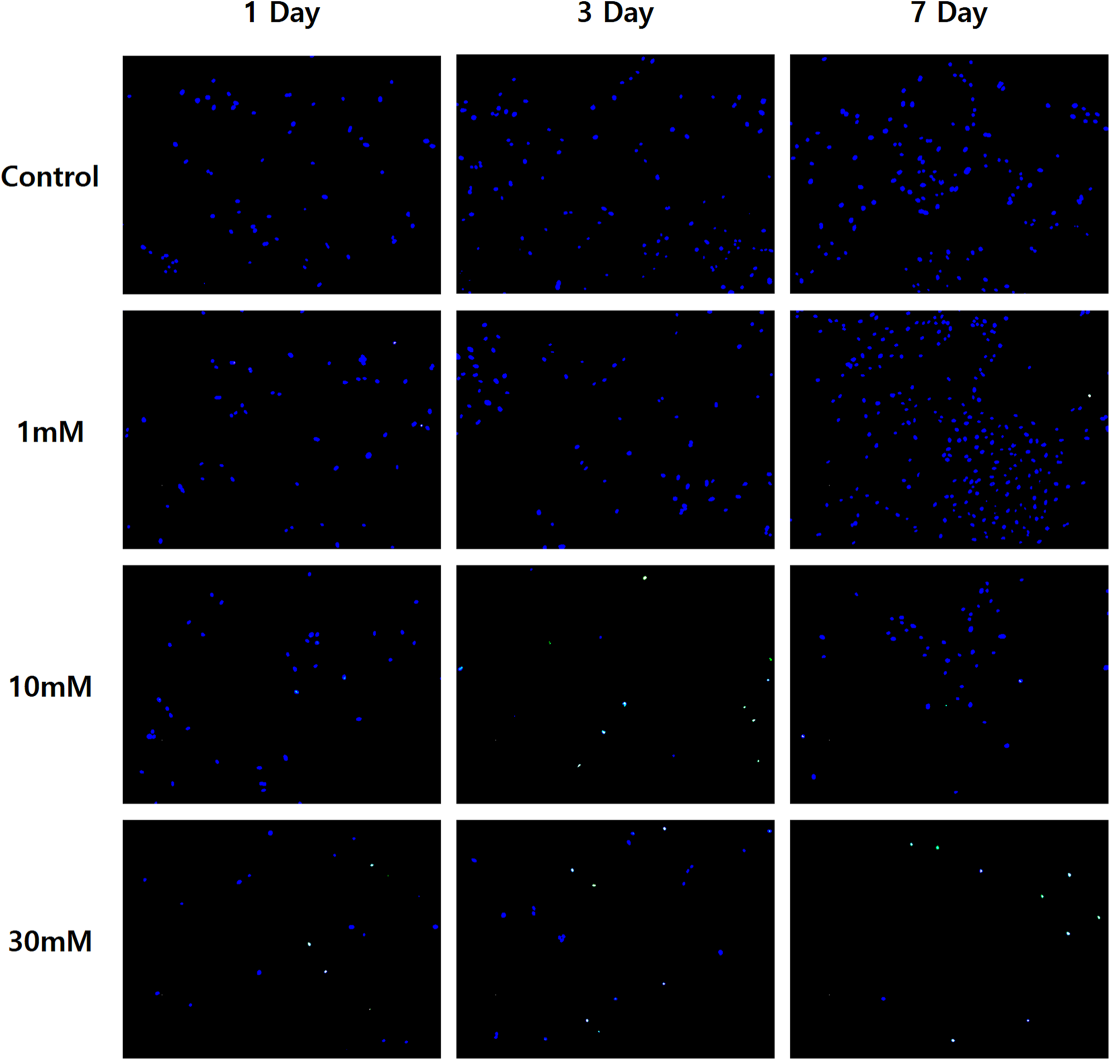
Fluorescence microscope images of nuclear counterstains with Hoechst staining and TUNEL assay. Proliferation (blue color) and apoptosis (small green spots) of MG-63 cell samples cultured with 0, 1, 10 and 30 mM of TSP for 1, 3 and 4 days were illustrated.

Fluorescence microscopy images of nuclear counter stains with TUNEL assay and Hoechst stain of 2D cultured cells were shown in Fig 1. Proliferations of control sample (the 1^st^ row images in Fig 1) and 1 mM TSP sample (the 2^nd^ row images in Fig 1) were increased with the incubation times: day 1, day 3 and day 7 (blue color in Fig 1). However, the number of blue-stained nuclei was dramatically decreased at high TSP dosages (≥10 mM) as shown in the 3^rd^ and 4^th^ rows in Fig 1. In addition, apoptosis of the cell specimen began to appear in the green dots at high TSP’s concentrations (≥ 10 mM).

### DNA Quantification vs. TSP Concentration

**Fig 2:**
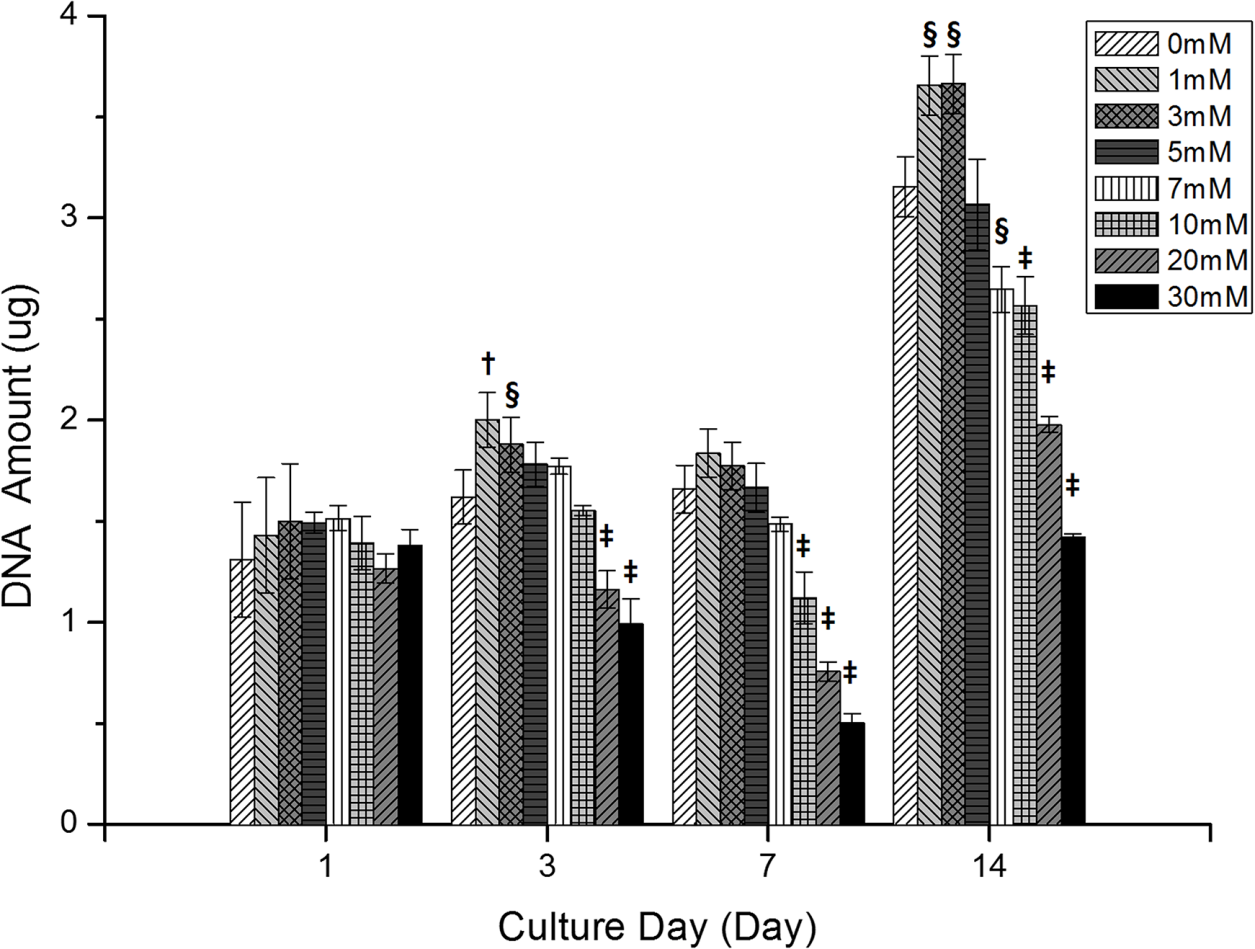
Graphical illustration of changes in DNA levels (cell proliferation) at days 1, 3, 7 and 14 with various TSP concentrations (0, 1, 3, 4, 7, 10, 20 and 30 mM) by DNA assay (n = 10). Symbols indicate significant differences in comparison with the TSP-free (0 mM) specimen (§: p<0.02, †: p<0.01, ‡:p<0.001).

Fig 2 shows the changes in DNA quantity (cell proliferation) at day 1, day 3, day 7 and day 14 during MG-63 cell culture in a three-dimensional environment, with various concentrations of TSP. There were no significant differences in the amount of DNA (p>0.05) among TSP concentrations at day 1. As shown in Fig 2, cells cultured with low concentrations of TSP had amounts of DNA similar to the 0 mM TSP control, but the amount of DNA in cells incubated with high concentrations of TSP decreased over the course of the experiment, again compared to the 0 mM TSP control. At day 3, the amount of DNA was significantly increased in cells cultured in low concentrations of TSP (1 mM; p<0.01, 3 mM; p<0.02), but decreased in samples with high TSP concentrations (p<0.001 for both 20 mM and 30 mM). At day 7, there was a significant reduction in the amount of DNA in samples with high TSP concentrations (10, 20 and 30 mM). After two weeks, DNA levels in all groups had increased considerably compared with those on day 7. The trends among the sample groups at day 14 were similar to those at other time points: DNA amount was increased in samples with low TSP concentrations (1 and 3 mM; p<0.02) and decreased in samples with high TSP concentrations (7 and 10 mM; p<0.02, 20 mM and 30 mM; p<0.001).

**Fig 3:**
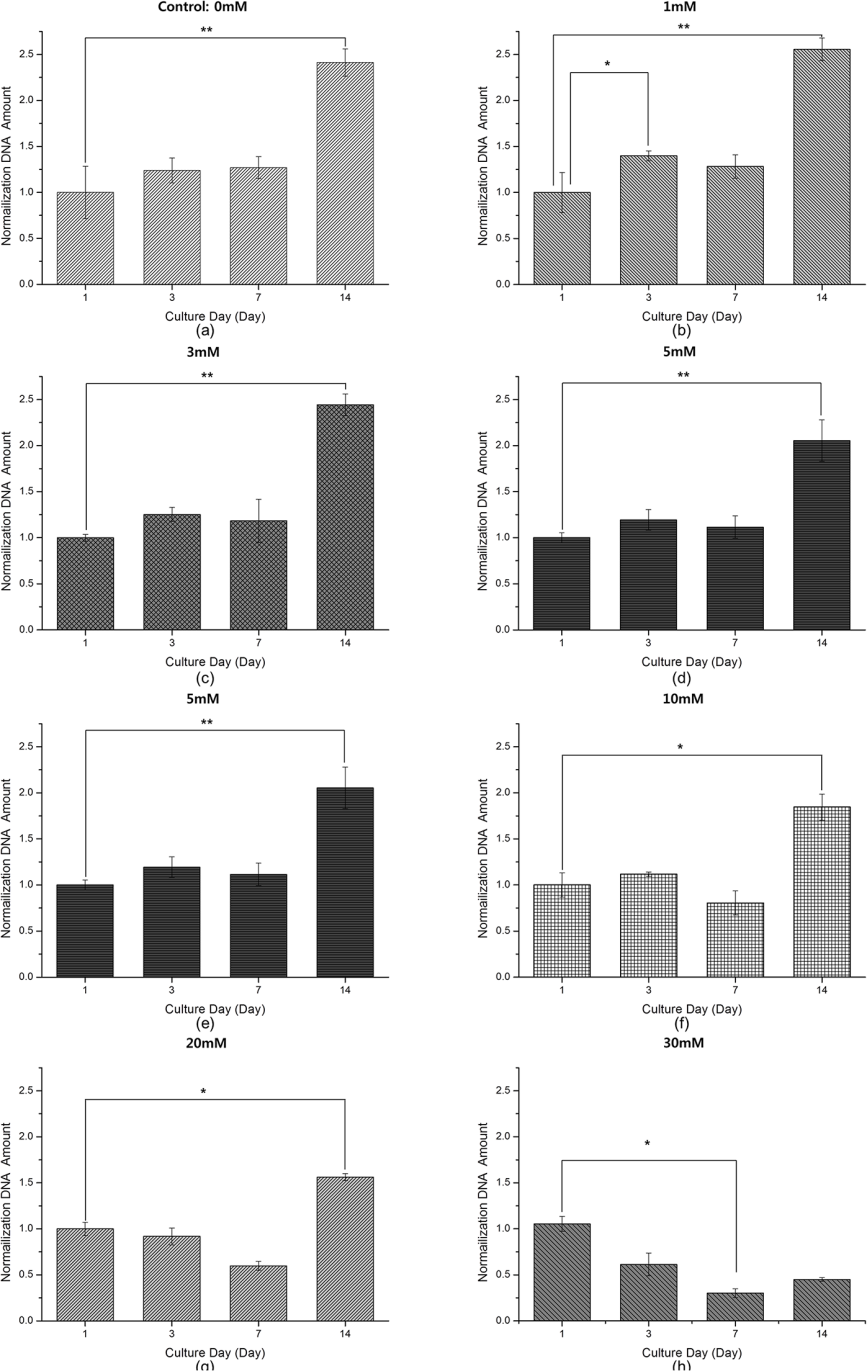
Changes in normalized DNA levels in samples of MG-63 cells during culture with various TSP concentrations: (a) 0 mM, (b) 1 mM, (c) 5 mM, (d) 10 mM, (e) 20 mM and (f) 30 mM. (n = 10, *: p<0.05, **: p<0.01).

The proliferation of MG-63 cells at each TSP concentration (0, 1, 5, 10, 20 and 30 mM) is shown in Fig 3. The DNA amounts in each graph were normalized to that of a TSP-free specimen. After one week of culture, there were no significant changes in the amount of DNA, irrespective of TSP concentration, with the exceptions of at 1 mM (increase at day 3), and 30 mM (decrease at day 7) (p<0.05). There was a marked, and statistically significant, increase in cell proliferation during the second week of culture (p<0.05 for 0, 10 and 20 mM TSP; p<0.01 for 1 and 5 mM). There was a visible trend towards decreased DNA amounts in the 30 mM TSP cultures at days 3, 7 and 14 (Fig 3(F)); however, the decline was significant only between day 1 and day 7.

### Cell Viability vs. TSP Concentration

**Fig 4:**
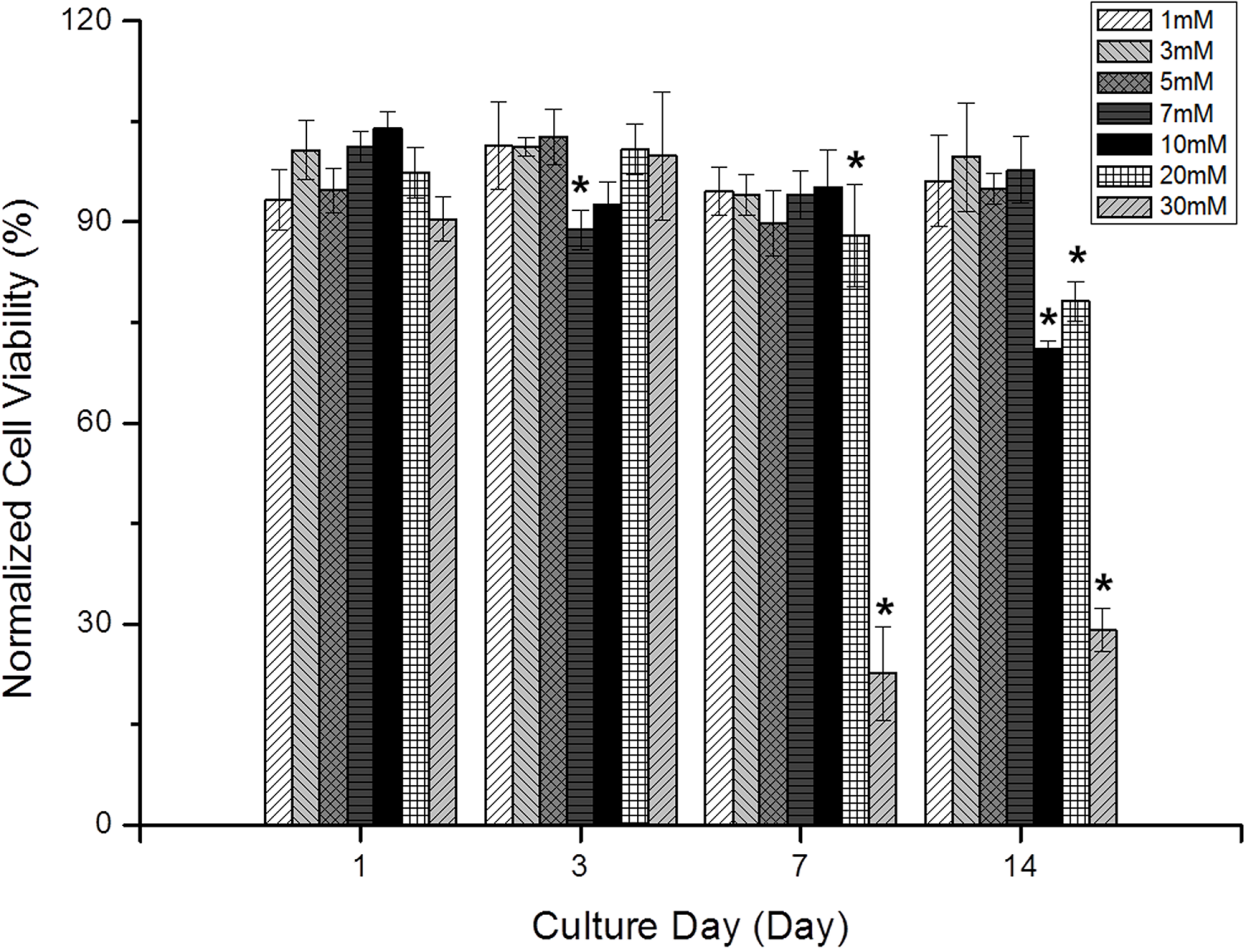
Graphical representation of the normalized changes in cell viability during two weeks of culture with various concentrations of the NMR reference reagent, TSP (n = 10 for each group, *: p<0.05).

The viability of MG-63 cells cultured in agarose gel with various TSP concentrations was measured at 1, 3, 7 and 14 days after cell seeding (Fig 4). All results were normalized to 0 mM TSP values (not shown) at each time point. There were no detectable changes in cell viability (≥84.9%) at day 3 for any TSP concentration. By day 7, cell viability had reduced to 21.5% in 30 mM TSP, and by day 14, viability was reduced to 70.1%, 77.0% and 28.7% in 10, 20 and 30 mM TSP, respectively. At days 7 and 14, the cell viability in 30 mM TSP decreased to below 21.5%. At day 14, cell viability also dropped below 70.1% in the 10 mM, 77.0% in 20 mM, and 28.7% in 30 mM TSP samples (Fig 4).

### Comparison between Proliferation and Viability

**Fig 5:**
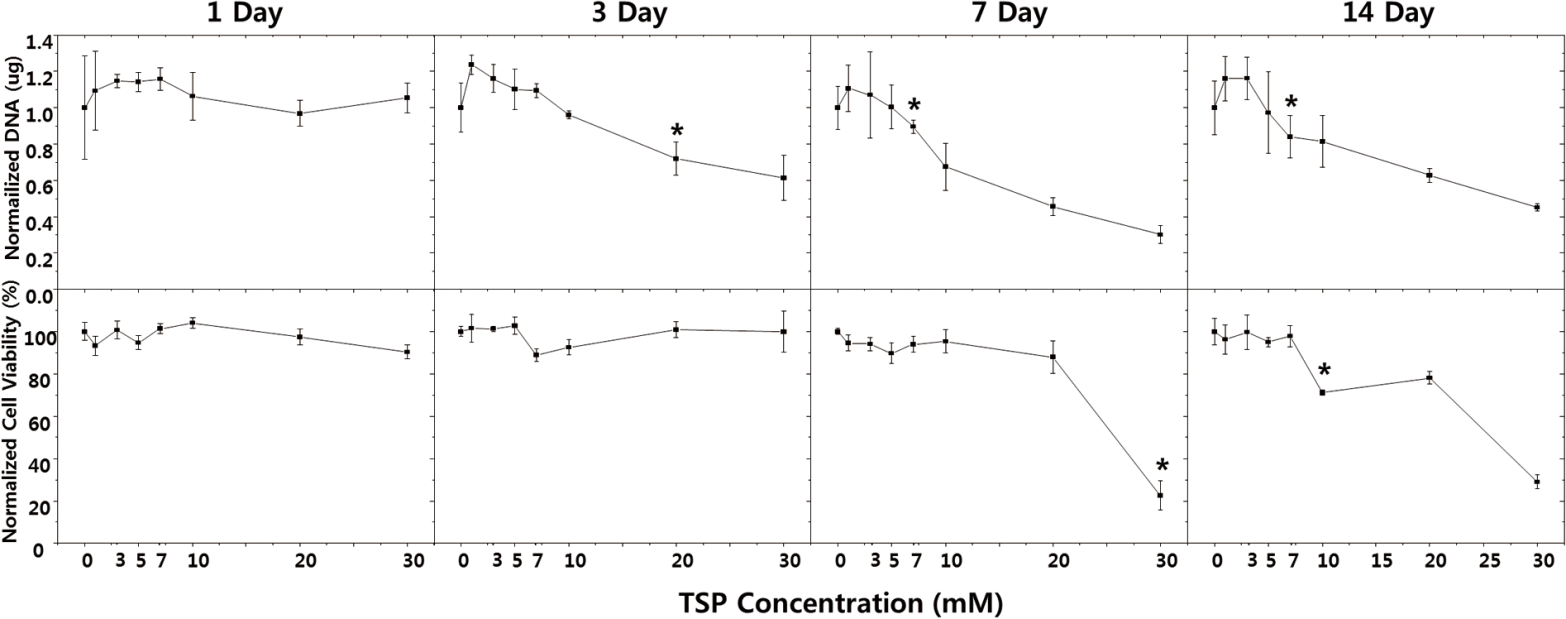
DNA amount and cell viability as a function of TSP concentration for each culture period. **DNA amounts at day 1 (a), day 3 (b), day 7 (c) and day 14 (d); cell-viability at day 1 (e), day 3 (f), day 7 (g) and day 14 (h).** All values are normalized to TSP-zero values at each time point. (* p<0.05).

In Fig 5, the decreases in DNA amounts (upper row) and cell viability (lower row) are shown as a function of medium TSP concentration at days 1, 3, 7 and 14. Both DNA amounts and cell viability are normalized to TSP-zero (0 mM) data values at each time point. DNA amounts were not significantly different at day 1 for all TSP concentrations (a), while they began to decrease as a function of TSP level; e.g., a 30% reduction from 1.66 to 0.5 μg at day 7. Slopes of the linear regression analyses were as follows: -0.25 (R^2^ = 0.15) at day 1 (a), -1.93 (R^2^ = 0.86) at day 3 (b), -2.79 (R^2^ = 0.93) at day 7 (c) and -2.25 (R^2^ = 0.89) at day 14 (d). Normalized cell viability values were not significantly changed at day 1 or day 3 for all TSP concentrations (e-f), while a TSP concentration-dependent decrease began at later time points; e.g., from 95% to 21% at day 7 (g) and from 99% to 29% at day 14 (h). Cell viability was not affected by TSP at day 1 (e) or day 3 (f), but was reduced at day 7 (g) and day 14 (h). Both DNA and cell viability decreased to below 50% in 30 mM TSP at days 7 and 14.

## Discussion and Conclusions

Conventionally, internal standard materials such as DSS and TSP in an NMR locking solvent (D_2_O) solution have been used as quantitative references for ^1^H NMR spectroscopy. A previous study reported that use of high concentrations of D_2_O (40% and 100%) in MG-63 cell culture medium, both during the NMR measurement and during the process of NMR sample preparation, is cytotoxic. The same study showed that using a low concentration of D_2_O (10%) for NMR did not alter cell viability during culture for up to two weeks [13]. In the present study, we evaluated the cytotoxicity of TSP in culture medium at concentrations greater than or equal to 1 mM during cell proliferation for two weeks.

The nature of the cytotoxicity observed in the cultured MG-63 cell line were examined by Hoechst staining / TUNEL assay (Fig 1). Increasing proliferation and no apoptosis were found in TSP-zero sample and 1 mM TSP sample during seven day culture period. From this results, authors believe that the toxicity and apoptosis are mainly induced by high dosages of TSP. As shown in Figs 2 and 3, the changes in MG-63 cell proliferation in a 3-D culture environment, as determined by quantifying DNA, suggest that TSP has concentration-dependent cytotoxic effects. DNA amounts increased significantly at day 14 compared with day 7 due to cell proliferation (Fig 2); i.e., cell proliferation during week 2 was increased compared to that in week 1. Significant (p<0.02) increases in the amount of DNA were found in samples with low TSP concentrations (≤3 mM) at day 3 and day 14 compared with that of TSP-zero samples. In contrast, high concentrations of TSP reduced the amount of DNA in the samples (p<0.01; ≥20 mM at day 3, ≥1 mM at day 7, ≥7 mM at day 14), as shown in Fig 1.

In Fig 3, DNA amounts at day 1 were set to 1.0, to which all other values were adjusted for comparison. The changes in the various TSP-concentration groups (Fig 3 (B)–3 (F)) can be compared with those of control (TSP-zero) samples (Fig 3 (A)). From Figs 2 and 3, increases in both TSP concentration and the duration of incubation in the presence of TSP reduced the amount of DNA. The greatest reduction in the DNA level—and therefore the greatest cytotoxic effect—was seen between TSP-zero samples (Fig 3(A)), and 30 mM TSP samples (Fig 3(F)), at days 7 and 14.

In the present study, cell viability (%) was measured as the ratio of viable cells to the total number of cells in the region-of-interest (ROI) in fluorescence images of the cell samples, as shown in Eq (1). This means that effects of cell proliferation, or any increase in the number of cells, were removed from cell viability measurements (Fig 4). Cell viability (>85%) remained constant regardless of TSP concentration for the first 3 days of culture, but high concentrations of TSP resulted in a reduction in cell viability at days 7 and 14; e.g., 30 mM TSP resulted in a cell viability of less than 30% at days 7 and 14.

The effects of TSP concentration on the proliferation (upper row, (a)-(d)) and viability (lower row, (e)-(h)) of MG-63 cells are shown in Fig 5. The DNA level was decreased from day 3 in cells cultured with high TSP concentrations (10 to 30 mM) (Fig 5 (B)–5 (D)). Samples with high TSP concentrations exhibited reduced cell viability beginning at day 7 (Fig 5(G)). Therefore, high TSP concentrations result in reduced cell proliferation at day 3, prior to the reduction in cell viability. The slopes of the proliferation and viability curves were also different. The reduction in the rate of proliferation between 20 and 30 mM was much lower compared to that between day 7 and day 14. Similar findings were reported by Medina et al. [17], who suggested that cellular mechanisms may be involved in the delayed cytotoxicity following exposure to drugs, and that mtDNA content is a significantly more sensitive measure of toxicity than is cell viability. [18]

In conclusion, the cytotoxicity of TSP to 3D cultured MG-63 cells was assessed by quantifying cell proliferation and viability. High concentrations of TSP (10 to 30mM) reduced cell proliferation and viability rates; however, no significant differences were observed for low TSP (<10 mM) concentrations. Therefore, the cytotoxicity of low concentrations of TSP in culture media is likely negligible for up to two weeks post-exposure. In the future, mechanisms of cytotoxicity due to the TSP should be identified. Also, it is necessary to study regarding the optimal concentration of TSP in accordance with the cell types.
